# Honeycomb-like MnO/C hybrids with strong interfacial interactions for aqueous zinc-ion batteries†

**DOI:** 10.1039/d5ra00089k

**Published:** 2025-02-24

**Authors:** Lin Li, Zhongcai Zhang, Yuan Ge, Ya Zhao, Wenru Wu, Xianliang Meng, Jiaxin Fan

**Affiliations:** a School of Chemical Engineering and Technology, China University of Mining and Technology Xuzhou 221116 China meng27@cumt.edu.cn; b School of Chemistry and Materials Engineering, Liupanshui Normal University Liupanshui Guizhou 553000 China fmf_fjx@lpssy.edu.cn; c College of Environmental and Chemical Engineering, Dalian University Dalian 116622 Liaoning China

## Abstract

Aqueous zinc-ion batteries (AZIBs) have garnered significant attention for large-scale energy storage applications due to their high theoretical capacity, low cost, and inherent safety. However, the absence of cathode materials exhibiting superior electrochemical performance severely impedes their further development. In this study, we report a metal–organic framework (MOF)-derived honeycomb-like MnO/C hybrid as a high-performance cathode material for AZIBs. A facile synthesis method was employed to uniformly embed MnO nanoparticles within a carbon matrix, thereby forming a honeycomb-like structure with robust heterointerfaces. This unique architecture provides efficient pathways for ion and electron transport, significantly enhancing structural stability and electrochemical performance. The MnO/C hybrid exhibits a high discharge specific capacity of 388 mA h g^−1^ at a current density of 50 mA g^−1^ and demonstrates excellent cycling stability, with a capacity decay rate of only 0.01% per cycle over 1000 cycles at a high current density of 2000 mA g^−1^. Comprehensive material characterization and electrochemical testing reveal the underlying mechanisms responsible for the superior electrochemical performance. This work provides a new perspective on the development of high-performance manganese-based cathode materials for AZIBs.

## Abstract 2

Honeycomb-like MnO/C hybrids for high-performance aqueous zinc-ion batteries.

## Graphical abstract

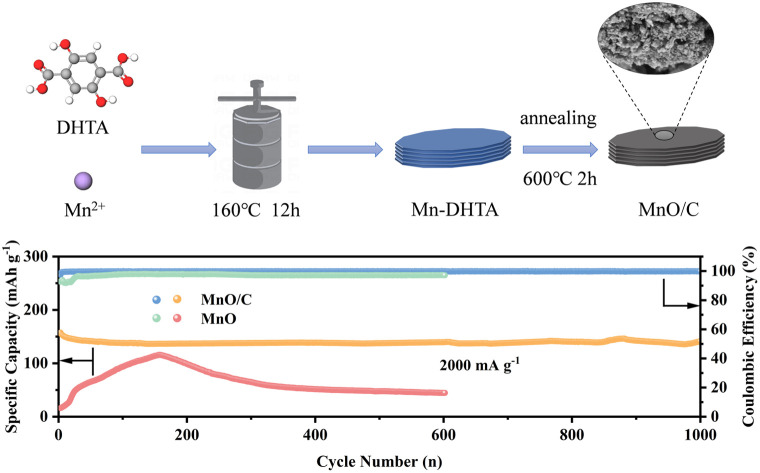

## 1 Introduction

The escalating global energy demand, coupled with increasing environmental concerns, necessitates the development of efficient and sustainable energy storage systems.^1,2^ Lithium-ion batteries (LIBs), widely adopted in various applications including solar and wind energy storage due to their high energy density, lightweight nature, and flexibility, suffer from drawbacks such as high cost, safety concerns, and environmental impact.^3^ Aqueous zinc-ion batteries (AZIBs) have emerged as a promising alternative energy storage technology, garnering significant attention in recent years due to their inherent advantages.^4–7^ Utilizing water as the electrolyte significantly enhances the safety profile of AZIBs compared to conventional organic electrolytes. Furthermore, the aqueous electrolyte simplifies the AZIB assembly process by eliminating the complex drying and explosion-proof measures required for LIBs, thereby improving manufacturing efficiency and convenience.^8,9^ The abundance and low cost of zinc metal provide economic viability for the large-scale commercialization of AZIBs. Moreover, the high theoretical capacity of zinc (820 mA h g^−1^) ensures a substantial energy density advantage, enabling AZIBs to store and release significantly more energy per unit mass compared to other alternatives.^10,11^

However, the performance of AZIBs is significantly constrained by the electrochemical activity and stability of electrode materials, particularly in the selection and optimization of cathode materials.^12,13^ A wide range of cathode materials for AZIBs has been reported, including manganese oxides, vanadium oxides, polyanionic compounds, Prussian blue analogs, organic cathode materials, and Chevrel phase compounds.^14–17^ Among these, manganese oxides are considered highly promising due to their abundant reserves (ranked 12th in the Earth's crust), facile synthesis, high discharge specific capacity (up to 250 mA h g^−1^), and elevated discharge plateau (above 1.4 V).^18–20^ Nevertheless, their development is hindered by issues such as Mn^2+^ dissolution during cycling,^21^ structural degradation of manganese oxides,^22^ and poor intrinsic conductivity.^23^ To enhance the electrochemical performance of manganese oxides, researchers have explored various strategies, including defect engineering, doping, and the fabrication of carbon-based composites.^24–27^ For instance, Yu *et al.*^24^ employed defect engineering to simultaneously introduce nitrogen (N) doping and oxygen (O) vacancies into commercially available, low-cost MnO, thereby activating the chemically inert MnO and significantly enhancing its zinc-ion storage capability. Zhu *et al.*^25^ improved the performance of MnO cathodes in AZIBs by electrochemically inducing cationic defects. Furthermore, carbon materials such as carbon nanotubes and graphene, known for their excellent conductivity, chemical stability, and mechanical strength, are widely utilized in diverse composites. Integrating manganese oxides with carbon materials forms composites that enhance conductivity, with carbon acting as a conductive network and structural support, thereby improving overall performance.^28,29^ Xiao *et al.*^30^ designed a MnO-CNT@C_3_N_4_ composite cathode using a hydrothermal and heat treatment strategy. The surface coating of CNTs effectively enhanced the conductivity of MnO, resulting in excellent rate performance (101 mA h g^−1^ at 3 A g^−1^) and high specific capacity (209 mA h g^−1^ at 0.8 A g^−1^) for the optimized MnO-CNT@C_3_N_4_. Li *et al.*^31^ synthesized MnO nanochains@graphene scrolls (MnO@NGS) *via* a modified hydrothermal process, leveraging graphene encapsulation and sufficient internal voids to achieve a reversible capacity of 288 mA h g^−1^ (at 0.1 A g^−1^) and an energy density as high as 367 mW h g^−1^. Zhang *et al.*^32^ synthesized a MnO/C@rGO composite by directly anchoring Mn-MOF onto reduced graphene oxide (rGO) sheets, followed by heat treatment under an Ar/H_2_ atmosphere. The composite exhibited good cycling stability (85.1% capacity retention after 1200 cycles at 2 A g^−1^) and rate capability (318.7 mA h g^−1^ at 0.2 A g^−1^). Li *et al.*^33^ prepared a MnO@C nanocomposite cathode, showcasing exceptional cycling performance in an electrolyte without Mn^2+^ additives, with a capacity decay of only 0.003% per cycle over 10 000 cycles. These findings demonstrate that incorporating carbon into composites enhances the conductivity of manganese oxides, strengthens the interfacial stability between electrodes and electrolytes, and consequently improves the energy efficiency and cycle life of the battery.

Inspired by these studies, we utilized manganese-based metal–organic frameworks (Mn-MOF) as precursors and leveraged the carbonization of their organic components during heat treatment to fabricate a MnO/C hybrid with a honeycomb structure, eliminating the necessity for additional carbon sources. This approach not only streamlines the preparation process but also efficiently employs the organic ligands in MOFs as carbon sources, thereby reducing costs and enhancing the material's sustainability. In the resulting hybrid, MnO nanoparticles are uniformly embedded within the honeycomb structure of the carbon matrix, establishing a robust heterointerface. The honeycomb structure not only offers efficient channels for ion and electron transport but also significantly enhances the structural stability and electrochemical performance of the hybrid through strong interactions between MnO and the carbon matrix. Specifically, the MnO/C hybrid demonstrates a high discharge specific capacity of 388 mA h g^−1^ at a current density of 50 mA g^−1^. It maintains a high capacity retention of 90% after 1000 charge–discharge cycles, with a capacity decay rate of only 0.01% per cycle. Furthermore, to gain a deeper understanding of the electrochemical behavior, we employed various spectroscopic techniques, including XRD, SEM, and XPS, to comprehensively analyze the structural characteristics and reaction mechanisms of the material. This analysis revealed a synergistic effect between MnO and the carbon matrix, enhancing both reactivity and structural stability. These findings offer a significant theoretical foundation and experimental basis for the development of novel high-performance MnO/C hybrid cathodes for AZIBs.

## 2 Experimental section

### 2.1 Preparation of Mn-MOF precursor

**Fig. 1:**
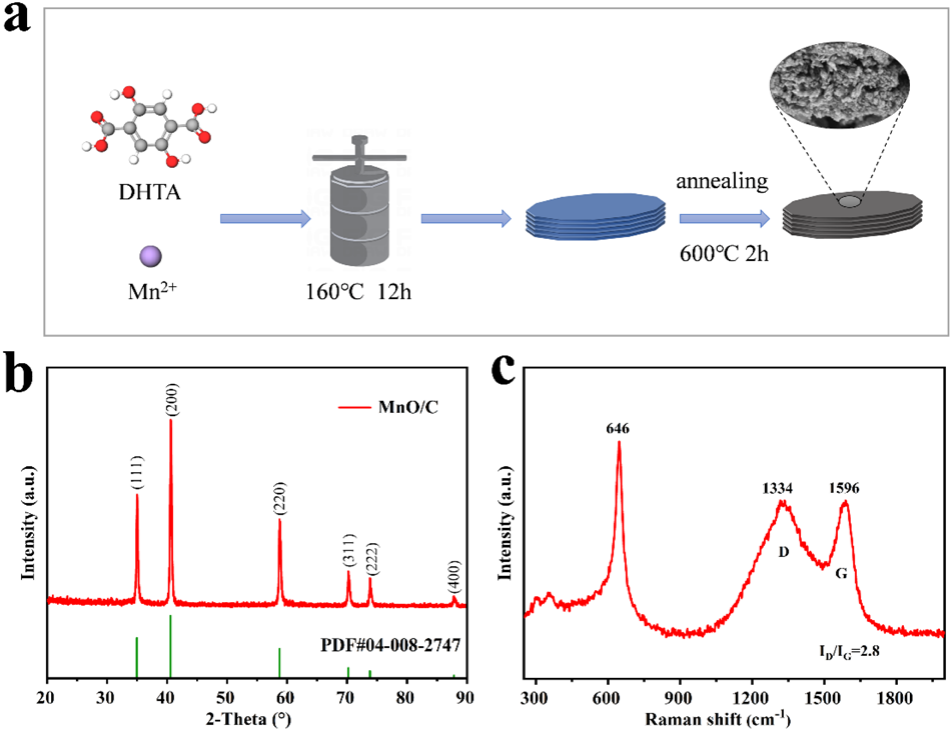
(a) A schematic illustration of the MnO/C hybrid material preparation process, (b) the XRD pattern, and (c) the Raman spectrum.

All chemicals used were of analytical grade and did not require further purification. MnCl_2_·4H_2_O (analytical grade, 99.0% purity), 2,5-dihydroxyterephthalic acid (DHTA, analytical grade, 98.0% purity), *N*,*N*-dimethylformamide (DMF, analytical grade, 99.5% purity), and ethanol (analytical grade, 99.5% purity) were purchased from Markin Company. The commercially available MnO electrodes were purchased from Xindu Metal Materials Company. The detailed preparation procedure is illustrated in Fig. 1a. First, 1.425 g of MnCl_2_·4H_2_O and 0.95 g of DHTA were weighed and added to 80 mL of DMF. The mixture was stirred at room temperature for 1 h until completely dissolved. Subsequently, the mixture was transferred to a 150 mL Teflon-lined stainless steel autoclave and maintained at 160 °C for 12 h. After the reaction, the Mn-MOF solid was filtered and washed three times with ethanol. The product was then dried in a vacuum oven at 70 °C for 10 h, yielding yellow Mn-MOF crystals.

### 2.2 Preparation of MnO/C

The obtained Mn-MOF precursor was heated to 600 °C at a rate of 4 °C min^−1^ under an argon atmosphere and maintained at this temperature for 2 h, followed by natural cooling to obtain MnO/C.

### 2.3 Material characterization

The crystal structure of the samples was determined using X-ray diffraction (XRD) patterns recorded with a Shimadzu 6100 X-ray diffractometer. X-ray photoelectron spectroscopy (XPS) analysis was conducted using a Thermo Scientific K-Alpha XPS instrument to determine the surface valence states and chemical composition. The morphology of the samples was characterized using a ZEISS Sigma 300 scanning electron microscope (SEM) and a Talos F200C transmission electron microscope (TEM). Raman spectroscopy was conducted using a Thermo Scientific DXR Raman spectrometer with a 633 nm excitation wavelength. Fourier transform infrared (FTIR) spectra were obtained using a Bruker INVENIO FTIR spectrometer. The pore size distribution and specific surface area were analyzed by N_2_ adsorption/desorption using a Micromeritics ASAP 2020 instrument. The Brunauer–Emmett–Teller (BET) equation and Barrett–Joyner–Halenda (BJH) model were used to calculate the specific surface area and pore size distribution, respectively. The carbon content of the samples was quantified by thermogravimetric analysis (TGA) using a Mettler TGA2 analyzer at a heating rate of 10 °C min^−1^ from room temperature to 700 °C.

### 2.4 Electrochemical measurements

Electrochemical performance was evaluated using CR2032 coin-type cells. The electrolyte consisted of a 2 M aqueous solution of Zn(CF_3_SO_3_)_2_. A zinc foil with a thickness of 50 μm was utilized as the anode. A glass fiber separator was employed. The cathode was fabricated by combining 80 wt% active material, 10 wt% Super P carbon black, and 10 wt% polyvinylidene fluoride (PVDF) in *N*-methyl-2-pyrrolidone (NMP) to form a homogeneous slurry, which was subsequently coated onto 50 μm thick carbon paper and dried at 70 °C for 2 h. The dried carbon paper was then cut into disks with a diameter of 1.4 cm. The active material loading on the cathode ranged from 0.9 to 1.4 mg cm^−2^. Electrochemical characterization was carried out using a VersaSTAT 3 electrochemical workstation. Cyclic voltammetry (CV) was performed at scan rates ranging from 0.2–3 mV s^−1^ within a potential window of 0.7–1.85 V to investigate the electrochemical behavior. Electrochemical impedance spectroscopy (EIS) measurements were performed over a frequency range from 100 kHz to 0.01 Hz to analyze the impedance characteristics at the electrode interface and elucidate the dynamic information regarding charge transfer and mass transport processes.

## 3 Results and discussion

### 3.1 Material characterizations

A honeycomb-like MnO/C hybrid with strong interfacial interactions was successfully synthesized through a hydrothermal reaction followed by annealing, as shown in Fig. 1a. Initially, a Mn–DHTA MOF material was prepared as a precursor for the MnO/C hybrid through a simple hydrothermal reaction. Subsequently, annealing the Mn–DHTA precursor under a nitrogen atmosphere yielded the MnO/C hybrid. The XRD pattern of the MnO/C hybrid (Fig. 1b) exhibits six distinct diffraction peaks at 34.9°, 40.6°, 58.7°, 70.2°, 73.8°, and 87.8°, corresponding to the (111), (200), (220), (311), (222), and (400) crystal planes of cubic *Fm*3̄*m* MnO (PDF #04-008-2747).^33,34^ The Raman spectrum (Fig. 1c) shows a characteristic MnO peak at 646 cm^−1^, attributed to the symmetric stretching vibration of the Mn–O bond. Two prominent peaks at 1334 cm^−1^ and 1596 cm^−1^ correspond to the D and G bands of carbon materials, respectively, confirming successful carbon hybridization. The D band is typically associated with defects and disordered structures in carbon materials, whereas the G band reflects the degree of graphitization. A higher *I*_D_/*I*_G_ ratio indicates greater defect density. On one hand, these defects create localized regions with higher electron density and reactivity, which serve as active sites for electrochemical reactions, thereby promoting faster charge transfer. On the other hand, the defect sites enhance the material's ability to adsorb ions from the electrolyte, which is crucial for the ion intercalation process in energy storage devices. In the MnO/C hybrid, *I*_D_/*I*_G_ = 2.8, suggesting that the material can provide more active sites during charge–discharge processes.^35^ FTIR analysis (Fig. S1†) of the precursor Mn–DHTA MOF sample reveals peaks at 1492–1349 cm^−1^ corresponding to the symmetric stretching vibration of –COO–, indicating successful coordination between Mn^2+^ ions and DHTA^2−^ ligands. Peaks at 1645–1598 cm^−1^ are attributed to the asymmetric stretching vibration of –COO–, and the peak at 780 cm^−1^ corresponds to the C–H bending vibration of the benzene ring. After pyrolysis, the FTIR spectrum of the MnO/C material shows significant changes. The disappearance of the organic peaks associated with the benzene ring, along with the appearance of a C=C stretching vibration peak at 1610 cm^−1^, originating from sp^2^ hybridized carbon atoms, further confirms the formation of the carbon layer. The two characteristic peaks observed at 518 cm^−1^ and 611 cm^−1^ are assigned to the lattice vibrations of Mn–O.^36^ The N_2_ adsorption–desorption isotherm of the MnO/C hybrid displays a clear hysteresis loop (Fig. S2†), indicating a type IV isotherm. This suggests the presence of a hierarchical pore structure dominated by mesopores, primarily formed by slit-like pores resulting from the stacking of nanoparticles. The material shows a high specific surface area of 200.4 m^2^ g^−1^, and the pore size distribution, calculated using the BJH method (Fig. S2†), reveals an average pore diameter of 5.8 nm. The high specific surface area and moderate pore size provide abundant active sites and efficient ion transport channels for zinc-ion batteries, while also reducing volume expansion during the insertion and extraction of zinc ions.

**Fig. 2:**
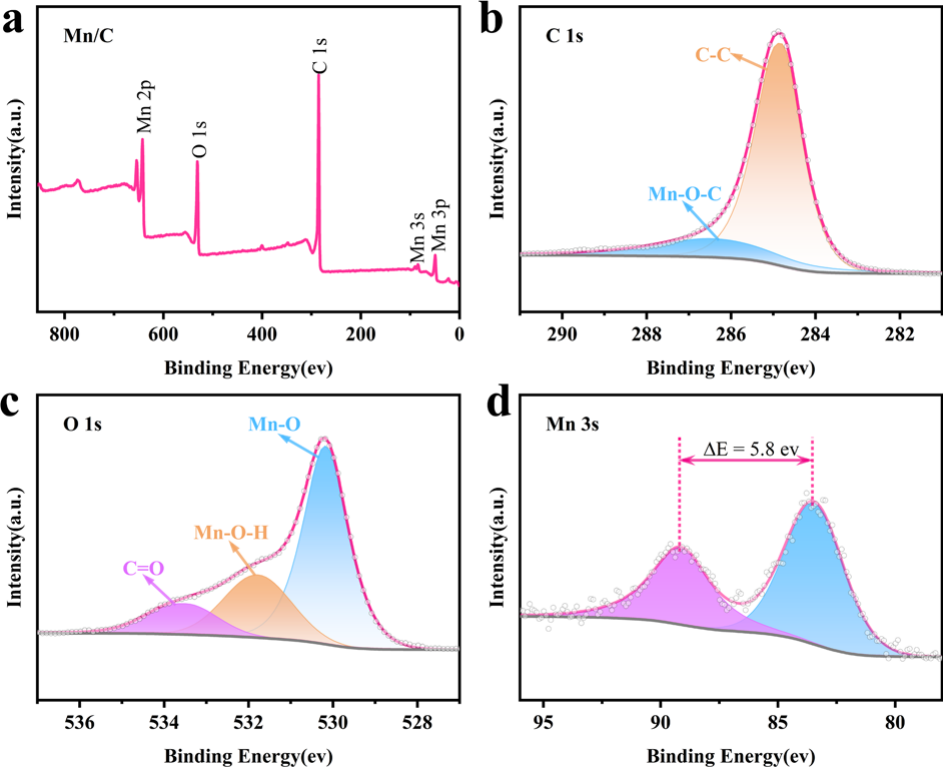
XPS spectra for the MnO/C hybrid: (a) survey spectrum and high-resolution spectrum of (b) C 1s, (c) O 1s, and (d) Mn 3s.

The chemical composition and valence states of the MnO/C hybrid were analyzed using X-ray photoelectron spectroscopy (XPS). Fig. 2a presents the survey spectrum of the MnO/C hybrid, displaying characteristic peaks corresponding to Mn 2p, O 1s, C 1s, Mn 3s, and Mn 3p. The high-resolution XPS spectrum of C 1s (Fig. 2b) reveals a dominant peak at 284.8 eV, attributed to sp^2^ and sp^3^ hybridized C–C bonds, indicating the presence of a carbon-based support structure. A peak at 286.4 eV is assigned to Mn–O–C bonds, confirming the formation of strong chemical bonds between MnO and the carbon matrix. This bonding is crucial for enhancing the electrochemical performance.^37^ The O 1s XPS spectrum (Fig. 2c) further substantiates the MnO–carbon interaction, resolving into three distinct peaks corresponding to Mn–O (530.2 eV), Mn–O–H (531.8 eV), and C=O (533.5 eV) bonds. The presence of these peaks indicates the formation of a heterogeneous structure between MnO and the carbon matrix, enhancing both structural stability and facilitating rapid electron transfer, thereby improving electrochemical performance.^32^ The Mn 2p XPS spectrum (Fig. S3†) shows two prominent peaks at 641.3 eV and 653.2 eV, assigned to Mn 2p_3/2_ and Mn 2p_1/2_, respectively. Furthermore, the Mn 3s XPS spectrum (Fig. 2d) reveals an energy separation of 5.8 eV between the two peaks, characteristic of Mn^2+^.^38,39^ In summary, XPS analysis reveals that the MnO/C hybrid exhibits a stable hybrid structure influenced by the carbon material, with Mn predominantly existing as Mn^2+^, thereby providing a theoretical basis for further enhancements in its electrochemical performance.

**Fig. 3:**
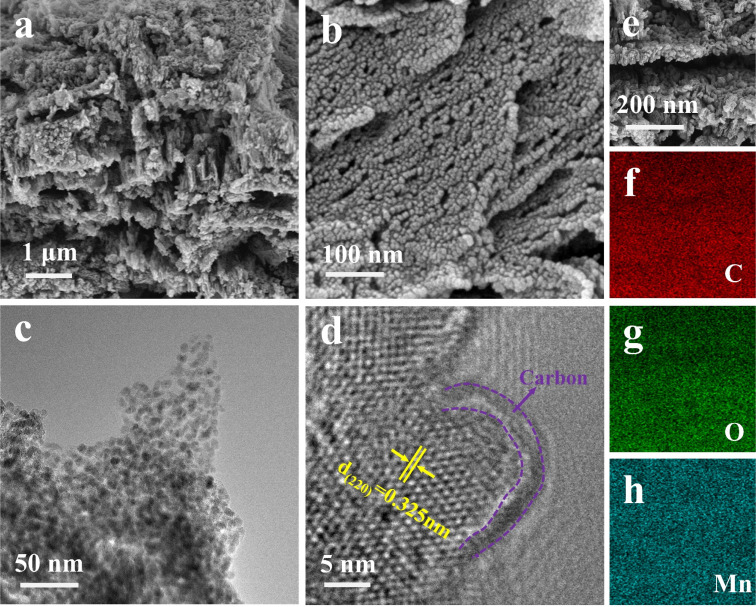
(a and b) SEM image. (c and d) Different resolutions of TEM and HRTEM images. (e–h) SEM image and corresponding elemental analysis.

The structure of MnO/C was characterized using scanning electron microscopy (SEM) and transmission electron microscopy (TEM). SEM images reveal that the initial Mn–DHTA precursor (Fig. S4†) consists of irregular cubic grains, which are densely packed, forming a macroscopic block-like structure. The grains possess smooth surfaces, with an average size of approximately 1 μm. As pyrolysis progresses, the organic ligands in Mn–DHTA decompose, releasing gases that create numerous voids within the material (Fig. 3a and S5†). Concurrently, the structure begins to reorganize, forming a honeycomb-like porous nanostructure (Fig. 3b). This reorganization significantly increases porosity and specific surface area, providing more contact area and transport pathways for zinc ions in the electrolyte, thereby enhancing the material's electrochemical activity. Further TEM analysis of the MnO/C hybrid (Fig. 3c and S5†) demonstrates that the MnO/C nanoparticles, approximately 10–15 nm in size, are uniformly embedded within the honeycomb-like structure formed by the carbon framework, thereby creating a heterogeneous structure with enhanced interactions. In the HRTEM image (Fig. 3d), a 2–4 nm thick carbon layer is clearly visible on the surface of the MnO/C nanoparticles. Additionally, an interplanar spacing of 0.325 nm is observed, corresponding to the (220) plane of MnO, which is consistent with XRD analysis. Elemental mapping *via* SEM (Fig. 3e–g) clearly shows that C, O, and Mn are evenly distributed throughout the material, further confirming the presence of a porous carbon layer and strong interacting heterogeneous interfaces. In addition, thermogravimetric analysis (TGA) was employed to determine the carbon content of MnO/C (Fig. S6†), which was found to be 12.3%.

### 3.2 Electrochemical properties of MnO/C

**Fig. 4:**
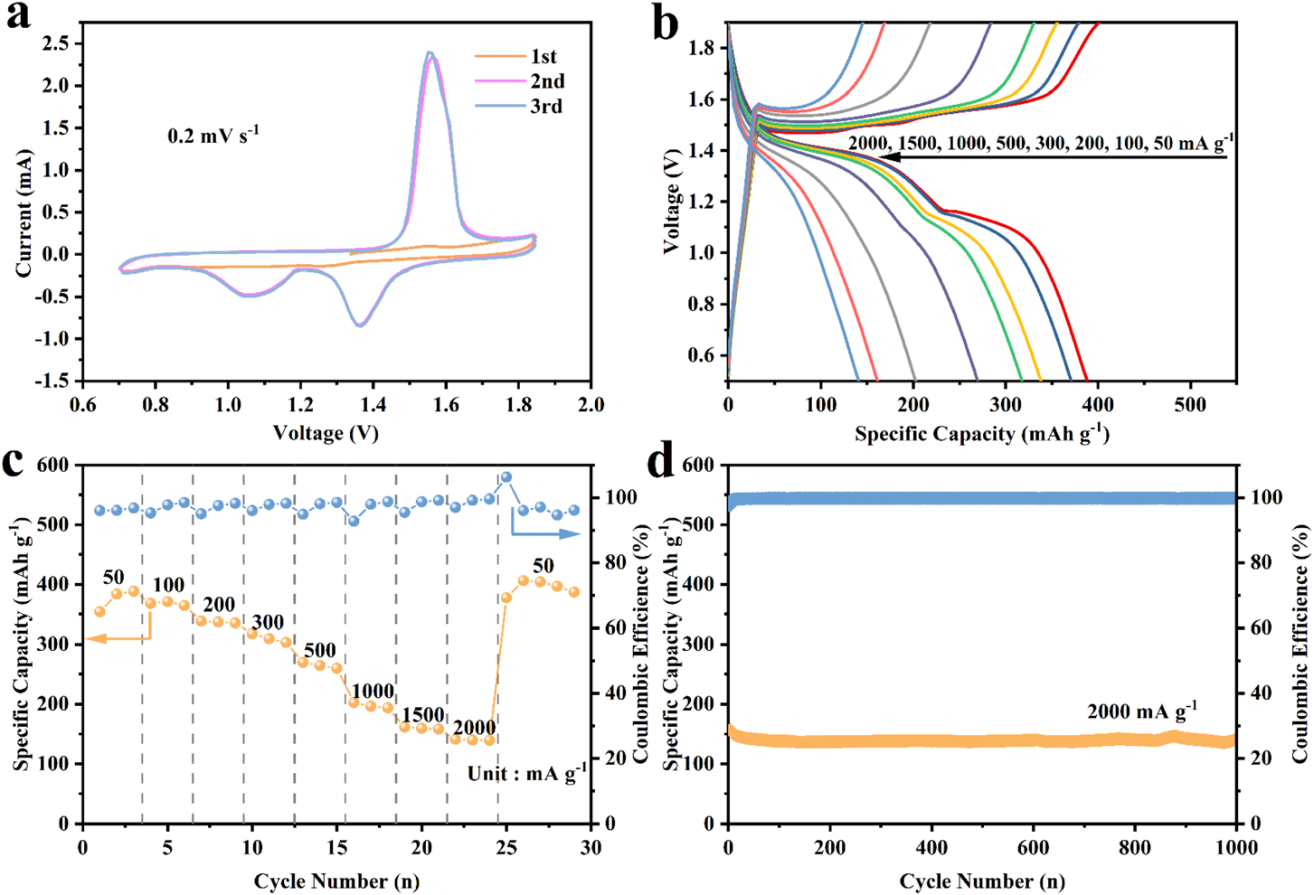
(a) CV curves at 0.2 mV s^−1^, (b) GCD curves of at different current densities, (c) rate performance, (d) cycle performances of the MnO/C at 2 A g^−1^.

To comprehensively evaluate the electrochemical properties of MnO/C hybrids, we assembled CR2025 coin cells utilizing MnO/C hybrids as the cathode material, zinc metal foil as the anode, and a 2 M Zn(CF_3_SO_3_)_2_ aqueous solution as the electrolyte. Initially, cyclic voltammetry (CV) tests were conducted for 1 to 3 cycles at a scan rate of 0.2 mV s^−1^ (Fig. 4a). No significant oxidation–reduction peaks were observed in the first cycle because the MnO in the cathode material undergoes a wetting process when it comes into contact with the aqueous electrolyte. However, the presence of the carbon layer modifies the surface properties of the cathode material and enhances the wetting behavior of the electrolyte on the electrode surface. As a result, distinct oxidation–reduction peaks become evident starting from the second cycle. Specifically, during the anodic sweep, a prominent anodic oxidation peak was detected at 1.56 V, attributed to the dissolution of Mn^2+^ ions and the partial oxidation of Mn^2+^ to Mn^3+^. In the cathodic sweep, two distinct cathodic reduction peaks appeared at 1.36 V and 1.05 V, corresponding to the insertion processes of H^+^ and Zn^2+^ ions, respectively. In the subsequent third cycle, the scans exhibited excellent overlap, indicating that the MnO/C electrode possesses outstanding electrochemical reversibility. Subsequently, galvanostatic charge–discharge (GCD) tests were performed on the MnO/C electrode, revealing charge–discharge plateaus consistent with the CV results (Fig. 4b). Additionally, as depicted in the rate performance graph (Fig. 4c), the cathode achieved a high discharge specific capacity of 388 mA h g^−1^ at an initial current density of 50 mA g^−1^. As the current density was incrementally increased to 100, 200, 300, 500, 1000, 1500, and even 2000 mA g^−1^, the discharge specific capacity gradually decreased to 371, 338, 318, 270, 203, 161, and 141 mA h g^−1^, respectively. However, this decline occurred in a smooth and orderly manner. Long-term cycling stability is also a critical parameter for evaluating the MnO/C cathode. At a current density of 200 mA g^−1^ (Fig. S7†), the capacity retention after 200 cycles was 97.3%. To our delight, under a high current density of 2000 mA g^−1^ (Fig. 4d), after 1000 repeated charge–discharge cycles, the electrode maintained a satisfactory capacity retention of 90%, with stable capacity curves. Additionally, the coulombic efficiency remained consistently around 100%. XRD and SEM characterizations were conducted on the electrode sheets before and after long cycling (Fig. S8 and S9†). The XRD patterns of the post-cycling electrodes show that the characteristic peak of MnO remains consistent with that observed before cycling, indicating that the material retains excellent crystalline structural stability throughout the prolonged cycling process, with no significant phase transition. The SEM images of the post-cycling electrodes demonstrate that the material's structure remains intact, with no signs of particle agglomeration, cracking, or structural collapse, suggesting that the electrode exhibits excellent mechanical stability and structural durability throughout prolonged electrochemical cycling. Compared to conventional commercially available MnO electrodes, the MnO/C hybrid cathode exhibits higher capacity and enhanced stability (Fig. S10†). The MnO/C hybrid cathode exhibits significantly superior electrochemical performance, not only highlighting its leading position in electrochemical characteristics but also further validating the substantial potential and strong competitiveness of MnO/C hybrids as advanced zinc-ion battery (AZIB) electrode materials. In addition, the comparison of electrochemical performance with other similar materials is provided in the ESI (Table S1).†

**Fig. 5:**
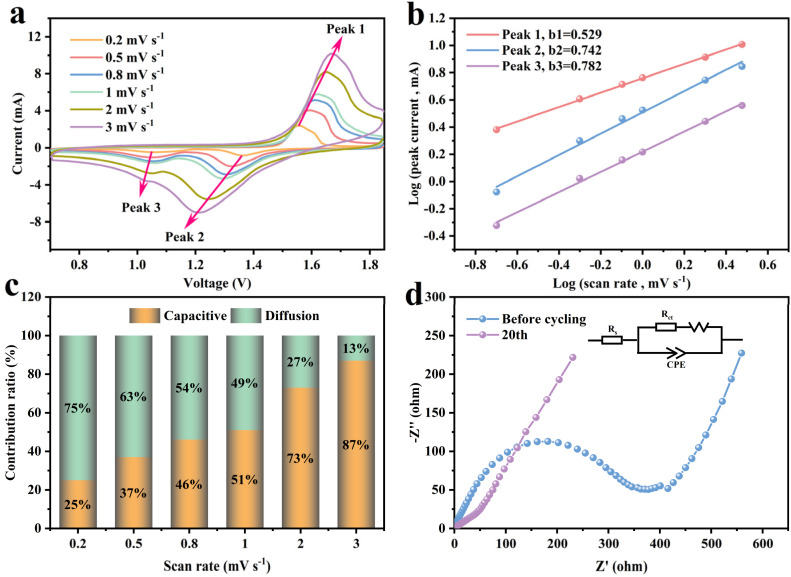
(a) CV curves of MnO/C at different sweep rates, (b) log(*i*) *vs.* log (*v*) plots of peaks in CV curves, (c) capacitive contribution, (d) EIS of the MnO/C.

To further elucidate the charge storage mechanism of the MnO/C cathode, we analyzed the cyclic voltammetry (CV) curves at various scan rates (Fig. 5a). The scan rates were varied from 0.2–3 mV s^−1^. The results indicated that as the scan rate increased, the peaks broadened progressively while maintaining their overall shape. Specifically, the anodic peak shifted slightly towards higher potentials, whereas the cathodic peak moved in the negative direction. During the cathodic sweep, two cathodic peaks emerged, Peak 2 corresponds to the insertion of H^+^, and Peak 3 corresponds to the insertion of Zn^2+^. This is attributed to H^+^ having a smaller ionic radius and faster migration rate compared to Zn^2+^, leading to H^+^ participation in the reaction first. It is well established that the relationship between peak current (*i*) and scan rate (*v*) is described by the empirical equation: *i* = *av*^*b*^, where *a* and *b* are variable parameters. The value of *b* ranges from 0.5 to 1.0. When the value of *b* is close to 0.5, it indicates a typical diffusion-controlled process, while the *b* value near 1.0 suggests a process controlled entirely by pseudocapacitance.^34,40^ By taking the logarithm of both sides of the equation and performing a linear fit (Fig. 5b), the value of *b* for the three redox peaks of the MnO/C electrode were determined to be 0.529 (Peak 1), 0.742 (Peak 2), and 0.782 (Peak 3), indicating that the process associated with Peak 1 is predominantly governed by the ion diffusion rate, whereas the redox reactions corresponding to Peak 2 and Peak 3 exhibit characteristics of both diffusion control and pseudocapacitive contributions, suggesting rapid electron transfer occurring at the electrode surface. Reportedly, the contribution of pseudocapacitance and diffusion behavior to the total charge storage conforms to the following equation:
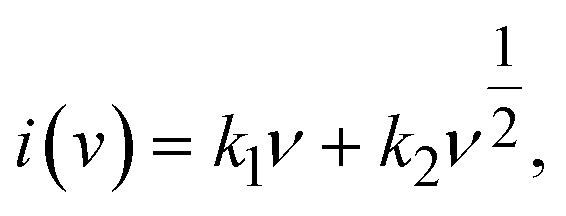
where *k*_1_*ν* represents the pseudocapacitive component.^41,42^ The pseudocapacitive contribution rates were evaluated at scan rates ranging from 0.2 to 3 mV s^−1^ (Fig. 5c), wherein, at a scan rate of 0.2 mV s^−1^, the pseudocapacitive contribution was 25%, increasing to 87% when the scan rate was elevated to 3 mV s^−1^. Electrochemical impedance spectroscopy (EIS) was used to compare the charge transfer resistance and ionic diffusion characteristics of the sample before and after 20 cycles (Fig. 5d). The semicircular arc observed in the high-frequency range corresponds to the charge transfer resistance (*R*_ct_), while the sloped linear portion represents the ion diffusion process within the electrode. Based on the equivalent circuit model (Fig. 5d), the internal resistance of the electrode (*R*_s_) was 0.892 Ω before cycling and 0.588 Ω after cycling, suggesting that the internal resistance of the aqueous electrolyte is inherently low. Initially, the *R*_ct_ was 297.1 Ω, but after 20 cycles, the electrode was rapidly activated, resulting in a significant reduction in *R*_ct_ to 13.7 Ω. Furthermore, the constant phase element (CPE) decreased from 105.6 μF to 11.1 μF, indicating that the active material on the electrode surface became more uniform during cycling. This analysis further supports the excellent diffusion and electron transfer properties of the MnO/C electrode.

**Fig. 6:**
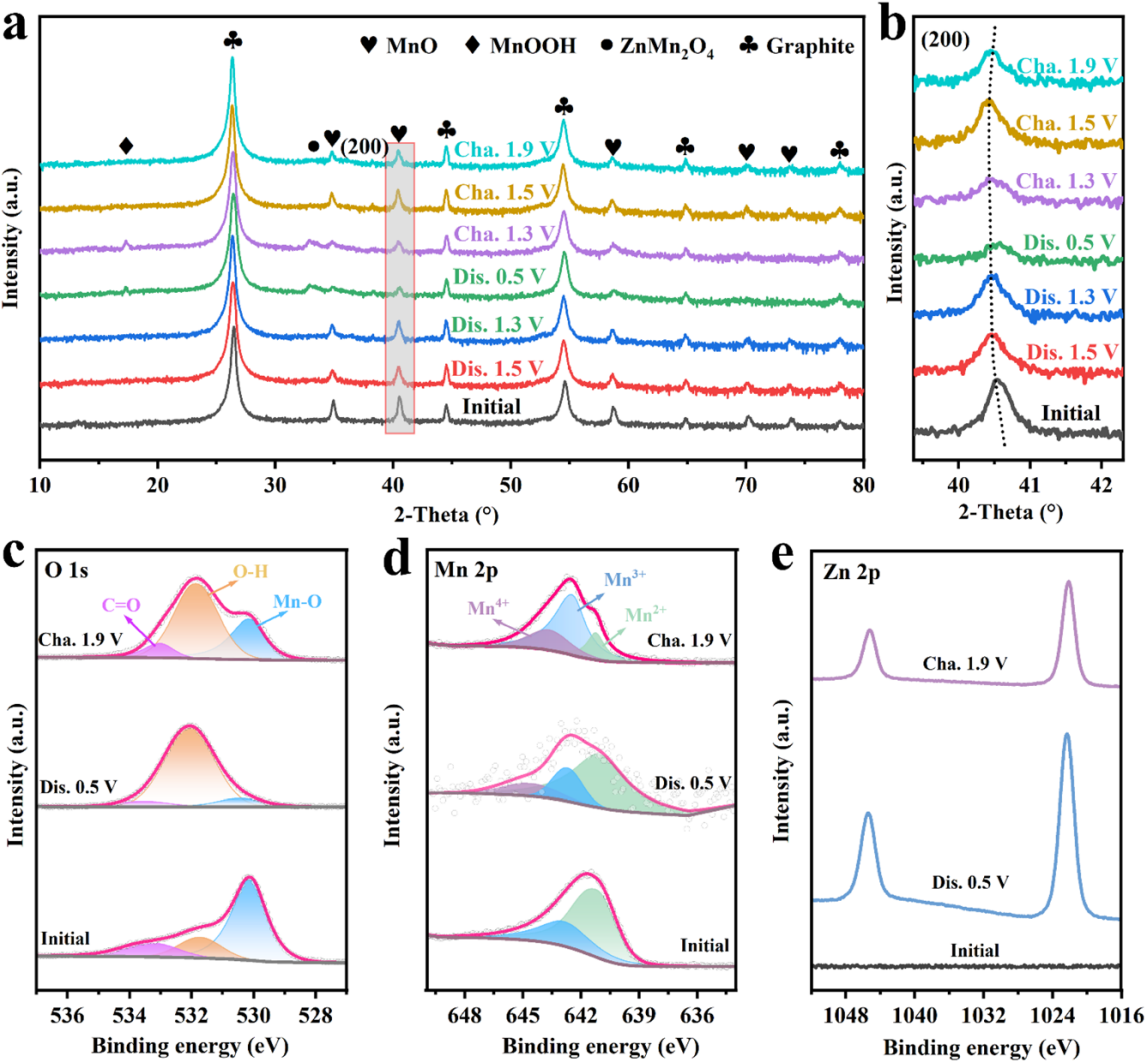
(a and b) *Ex situ* XRD patterns of the MnO/C cathode at different states. High-resolution XPS spectra: (c) O 1s, (d) Mn 2p and (e) Zn 2p at different states of the MnO/C cathode.

To further investigate the storage mechanism of the MnO/C cathode during the charge–discharge process, *ex situ* XRD and XPS characterizations were conducted. As shown in Fig. 6a and b, the XRD patterns under various charge–discharge states are presented. Overall (Fig. 6a), characteristic peaks of the MnO phase persist throughout the entire charge–discharge process, indicating the structural stability of the MnO/C cathode. Upon discharging to 0.5 V, a new weak diffraction peak appears at 17.1°, corresponding to the formation of MnOOH (PDF #74-1632) during the H^+^ insertion process. Additionally, around 33°, the formation of ZnMn_2_O_4_ (PDF #71-2499) due to Zn^2+^ insertion is observed.^43,44^ When charged to 1.3 V, these two new phases can still be observed. However, as charging progresses, the diffraction peaks of MnOOH and ZnMn_2_O_4_ disappear due to the gradual de-insertion of H^+^ and Zn^2+^ ions, which is consistent with the CV analysis. Furthermore, during the discharge process (Fig. 6b), a slight shift of the derivative peak of the (200) crystal plane of the MnO/C cathode towards lower angles is observed. This shift is attributed to the expansion of the lattice spacing caused by the insertion of H^+^ and Zn^2+^ ions. More importantly, the derivative peak of the (200) crystal plane gradually returns to its original position during the charging process, indicating that the MnO/C cathode material maintains structural stability and high reversibility during the insertion and de-insertion of H^+^ and Zn^2+^ ions. This is closely related to the scaffold structure of the carbon layers in the MnO/C heterostructure.^45^*Ex situ* XPS was further employed to analyze the surface valence states and composition of the MnO/C cathode under different states. Firstly, the O 1s XPS spectra (Fig. 6c) indicate that in the initial state, the intensity of the O–H characteristic peak is relatively low. As the discharge process progresses, the peak intensity of the O–H characteristic increases gradually, due to the formation of a significant amount of OH^−^ and H_2_O. Conversely, during the charging process, the intensity of O–H gradually decreases, which can be attributed to the decomposition of Zn salts. Secondly, examining the Mn 2p_3/2_ XPS spectra (Fig. 6d) reveals that Mn exists primarily in the Mn^2+^ state in the initial condition. Upon charging to 1.9 V, Mn^2+^ is oxidized to higher valence states Mn^3+^ and Mn^4+^, with the distribution of manganese valence states being Mn^2+^ 17.4%, Mn^3+^ 60.1%, and Mn^4+^ 22.5%. When discharged to 0.5 V, the distribution of manganese valence states changes to Mn^2+^ 71.8%, Mn^3+^ 16.6%, and Mn^4+^ 11.6%, indicating a significant increase in Mn^2+^ content and a notable decrease in Mn^4+^ content. This suggests a reversible conversion between Mn^4+^ and Mn^2+^. Additionally, as shown in Fig. 6e, no traces of Zn were detected in the Zn 2p XPS spectra in the initial state. Upon discharging to 0.5 V, two pairs of Zn 2p signals appear in the spectra, attributed to Zn^2+^ adsorbed on the cathode surface and Zn^2+^ embedded within the MnO/C structure. When charged to 1.9 V, a single pair of Zn 2p signals is observed, which is likely attributed to the adsorption of surface electrolytes, exhibiting lower intensity compared to the discharged state. In summary, the storage mechanism of MnO/C can be described by the following equations.

Cathode reaction:2MnO → MnO_2_ + Mn^2+^ + 2e^−^Mn^2+^ + 4OH^−^ ⇋ MnO_2_ + 2H_2_O + 2e^−^MnO_2_ + H_2_O + e^−^ ⇋ MnOOH + OH^−^Zn^2+^ + 2MnO_2_ + 2e^−^ ⇋ ZnMn_2_O_4_

Anode reaction:Zn ⇋ Zn^2+^ + 2e^−^

Energy density and power density are crucial parameters for assessing a battery's energy storage and output capabilities. Fig. S11† presents the Ragone plot of MnO/C. Compared to recently reported zinc-based rechargeable battery electrodes, including Mn_2_O_3_/Al_2_O_3_,^46^ Zn_3_V_2_O_7_,^47^ CNF-Zn-800,^48^ Ni–Zn,^49^ MnO/CC-N,^50^ and D-Fe–N/C,^51^ the MnO/C hybrid demonstrates significant advantages and shows great potential for the development of AZIBs.

## 4 Conclusions

In summary, this study successfully developed a novel MnO/C hybrid material using manganese-based MOF (DHTA) as a precursor. The resulting heterostructure between MnO and the carbon layer significantly enhances its electrochemical performance. The presence of the carbon matrix effectively improves ion and electron transfer processes, thereby increasing the structural stability of the MnO/C hybrid material. Leveraging this synergistic effect, the MnO/C hybrid achieved a high discharge capacity of 388 mA h g^−1^ at a current density of 50 mA g^−1^. Additionally, it exhibited excellent cycling stability, maintaining 90% of its capacity after 1000 charge–discharge cycles at a high current density of 2000 mA g^−1^, with a minimal capacity decay rate of only 0.01% per cycle. Furthermore, various characterization methods were employed to investigate the energy storage mechanisms of the electrode. These findings not only provide new insights for designing high-performance manganese-based cathode materials but also lay a solid foundation for the development of future cathode materials for zinc-ion batteries.

## Data availability

All data that support the findings of this study are included within the article (and any ESI files†).

## Author contributions

Lin Li: conceptualization, writing – original draft, writing – review & editing. Zhongcai Zhang: data curation. Yuan Ge: conceptualization, investigation. Ya Zhao: software. Wenru Wu: investigation. Xianliang Meng: supervision, writing – review & editing. Jiaxing Fan: resources, writing – review & editing, project administration.

## Conflicts of interest

There are no conflicts to declare in our work.

## Supplementary Material

RA-015-D5RA00089K-s001
