# Supplementary material for: Honeycomb-like MnO/C hybrids with strong interfacial interactions for aqueous zinc-ion batteries
Source: RSC Adv. 2025 Feb 24;15(8):5942–50. doi: 10.1039/d5ra00089k (PMC11848712; doi:10.1039/d5ra00089k)
Supplement: RA-015-D5RA00089K-s001 [file RA-015-D5RA00089K-s001.pdf]

Supplementary Information (SI) for RSC Advances.
This journal is © The Royal Society of Chemistry 2025

Honeycomb-like MnO/C Hybrids with Strong Interfacial Interactions for Aqueous Zinc-Ion Batteries

Lin Li [ab], Zhongcai Zhang [a], Yuan Ge [ab], Ya Zhao [c], Wenru Wu [b], Xianliang Meng[*a] and Jiaxin Fan[*b]

[a]School of Chemical Engineering and Technology, China University of Mining and Technology, Xuzhou 221116, China.

[b]School of Chemistry and Materials Engineering, Liupanshui Normal University, Liupanshui, Guizhou 553000, China.

[c]College of Environmental and Chemical Engineering, Dalian University, Dalian, 116622, Liaoning, China.

*Corresponding Author:

*E-mail: meng27@cumt.edu.cn (Xianliang Meng), fmf_fjx@lpssy.edu.cn (Jiaxin Fan)

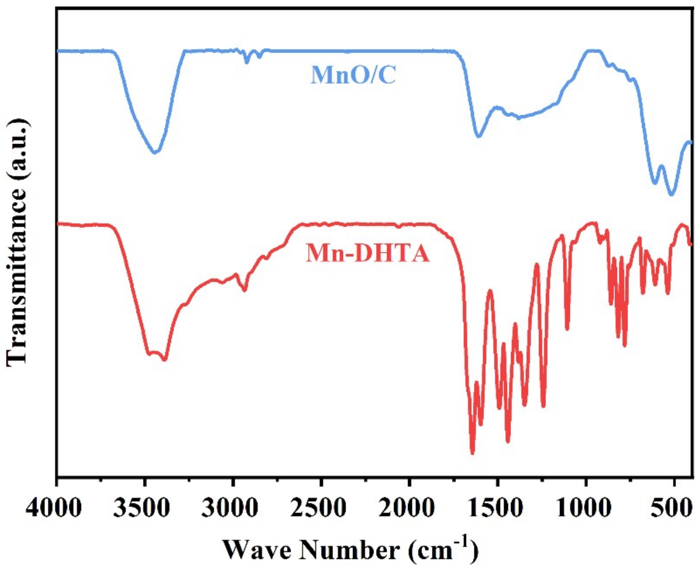

Fig. S1 FT-IR spectra of MnO/C and Mn-DHTA.

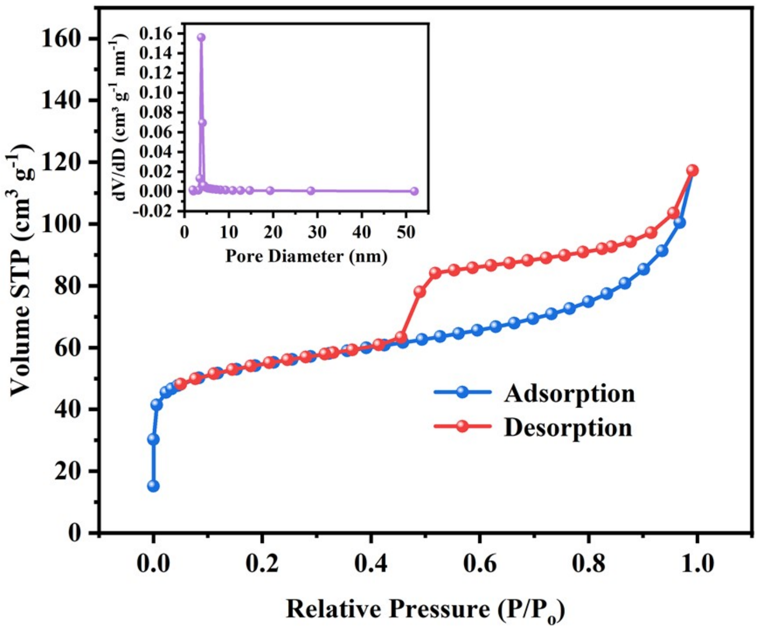

Fig. S2 Adsorption/desorption curve of MnO/C (the inset is pore size distribution curve).

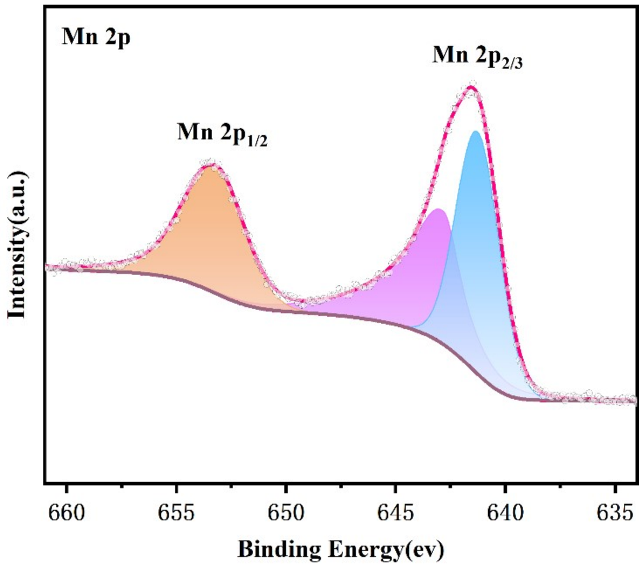

Fig. S3 XPS spectrum of Mn2p in MnO/C hybrid.

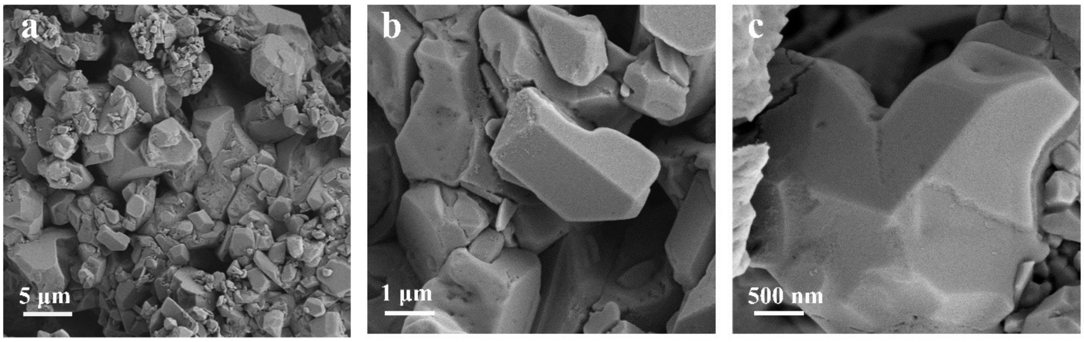

Fig.S4 SEM image of Mn-DHTA.

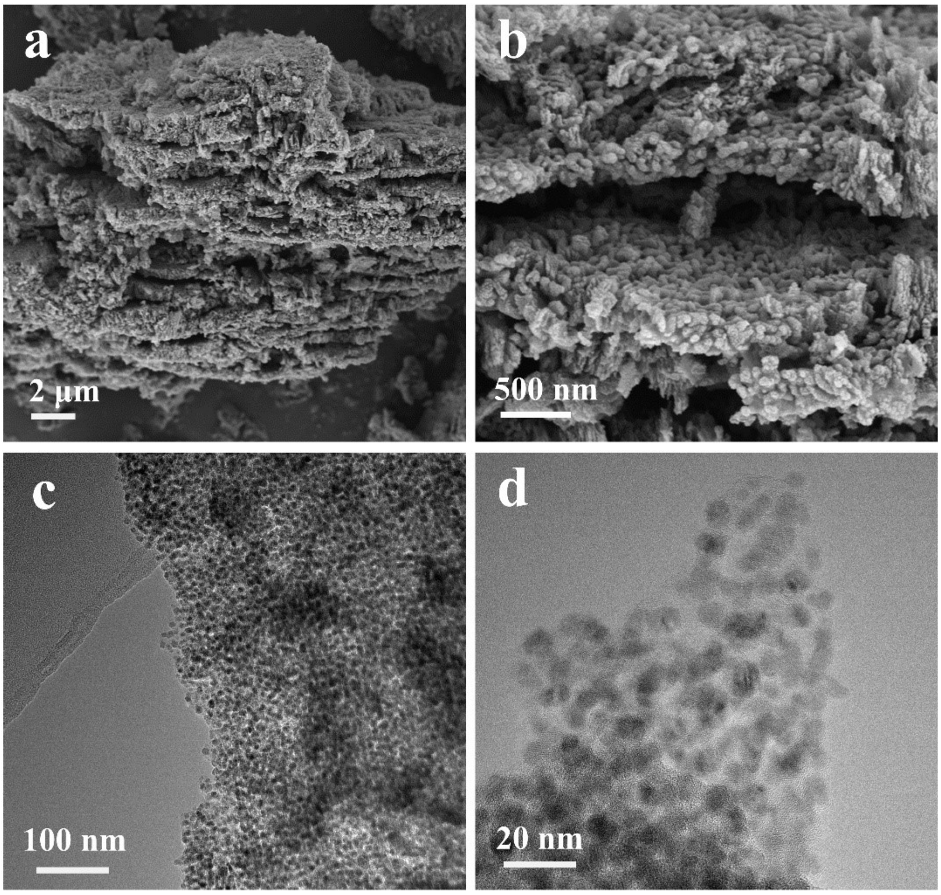

Fig. S5 (a-b) SEM image and (c-d) TEM image of Mn/O.

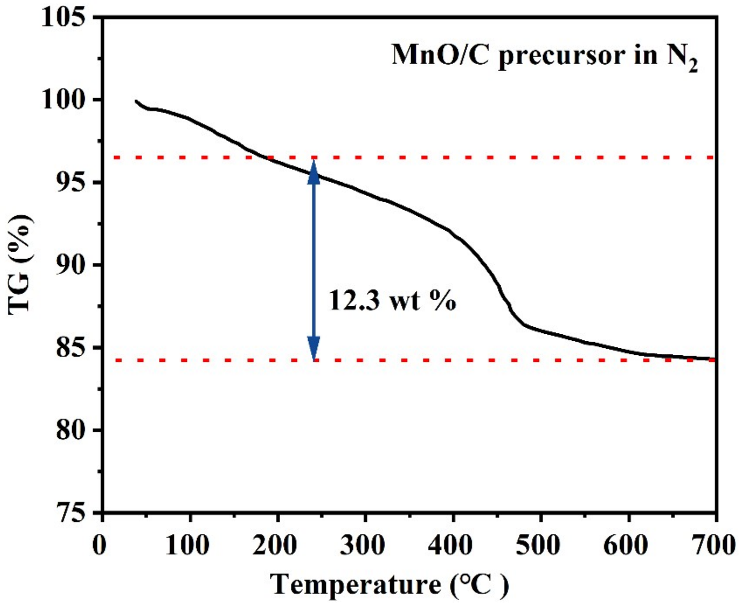

Fig. S6 TG curve of the precursor in N_2

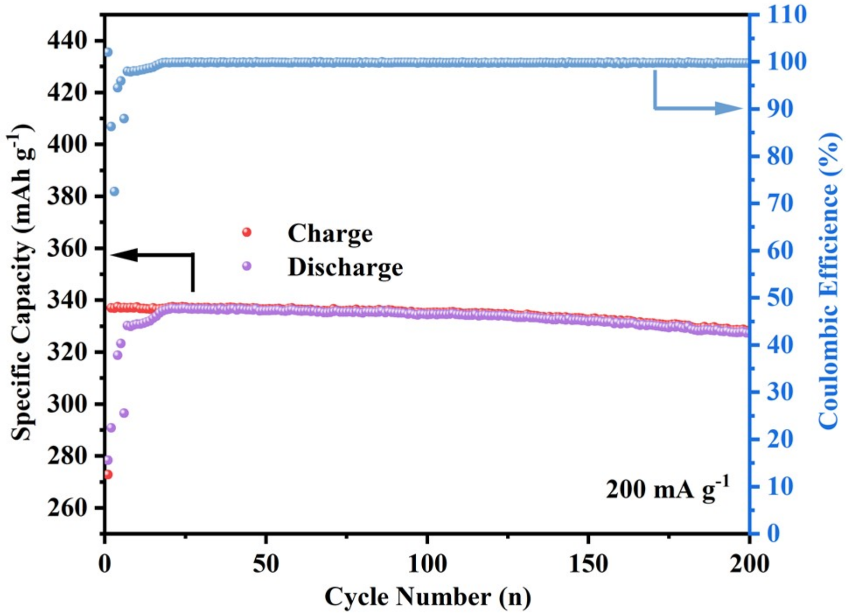

Fig. S7 Cycle performance of MnO/C at 200 mA g^{-1}.

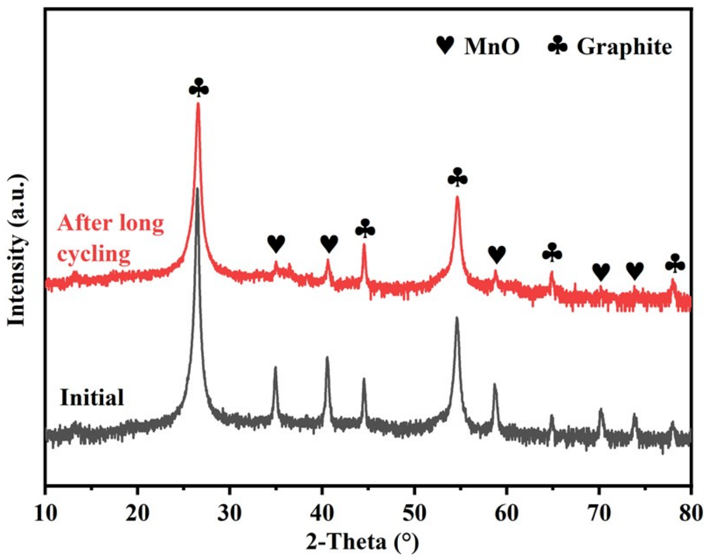

Fig. S8 XRD comparison before and after long-term cycling.

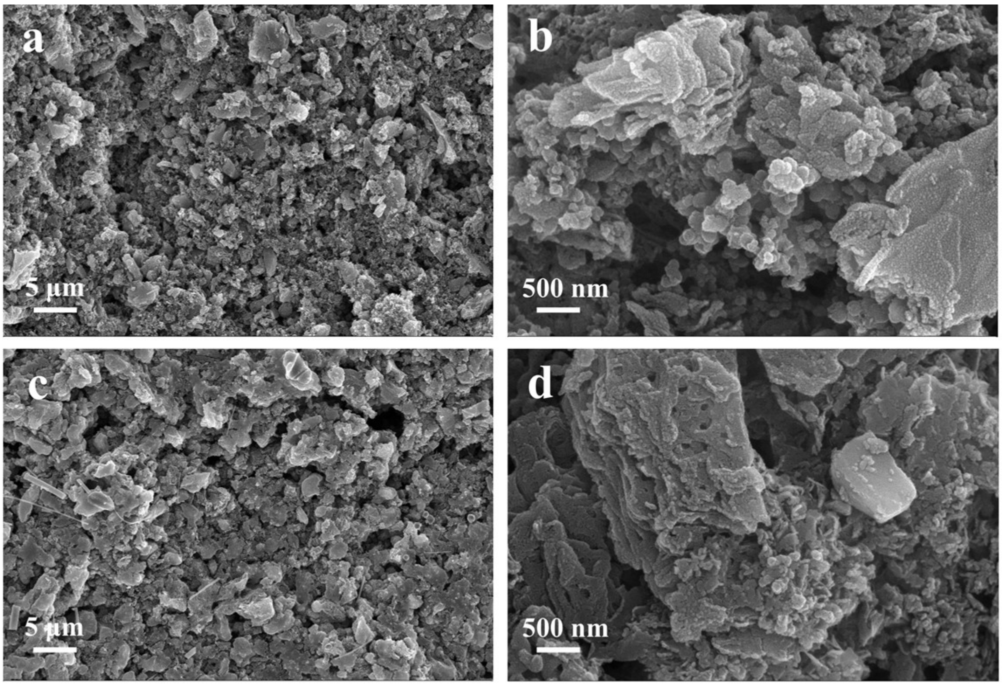

Fig. S9 (a-b) SEM images of MnO/C before cycling, (c-d) SEM images of MnO/C after long-term cycling.

Table S1. Electrochemical performance comparisons of the MnO/C hybrid with other recently reported manganese-based oxides cathode materials.

Cathodes	Current density A g^{-1}	Cycles numbers	Capacity retention	Ref.
**MnO/C**	0.2<br>2	300<br>1000	%<br>90.0%	**This work**
**α-Mn_2O_3**	2	2000	65.0%	[1]
**MnO_2/CC**	1	300	93.0%	[2]
**MnO_2@NC**	5	1000	83.0%	[3]
**CC@MnO_2 -RD_5**	2	1000	90.4%	[4]
**β-MnO_2**	0.2	200	75.0%	[5]
**Hausmannite Mn_3O_4**	1	800	65.0%	[6]
**Mn_2O_3/Al_2O_3**	1.5	1100	88.0%	[7]
**β-MnO_2 nanosheet**	2	2000	94.0%	[8]
**Mn_2O_3-MOF**	1	500	86.1%	[9]
**MnHCF**	0.1	70	87.1%	[10]
**MXene@MnO2**	2	3000	85.0%	[11]

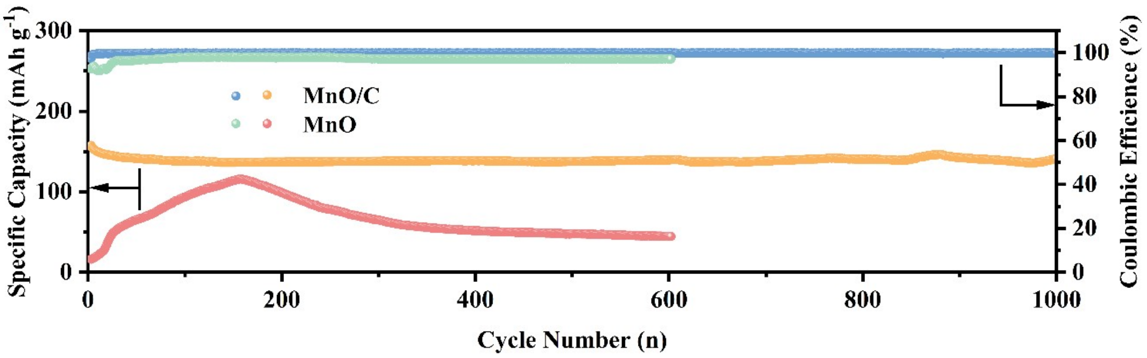

Fig. S10 Cycle performances of the MnO/C and MnO cathodes at 2 A g^{-1}.

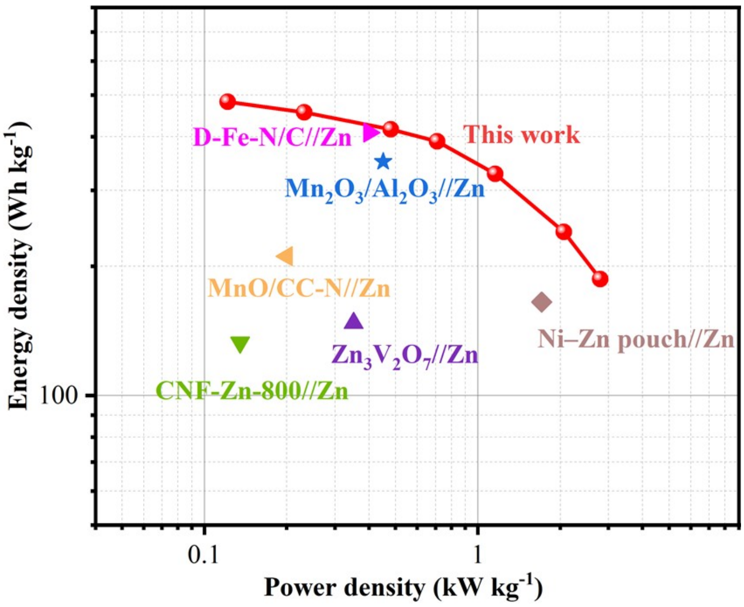

Fig. S11 The Ragone plots of MnO/C//Zn in comparison with those of other reported batteries.

References

1 B. Jiang, C. Xu, C. Wu, L. Dong, J. Li and F. Kang, *Electrochim. Acta*, 2017, **229**, 422-428.
2 F. Wu, X. Gao, X. Xu, Y. Jiang, X. Gao, R. Yin, W. Shi, W. Liu, G. Lu and X. Cao, *ChemSusChem*, 2020, **13**, 1537-1545.
3 Y. Zhang, Y. Liu, Z. Liu, X. Wu, Y. Wen, H. Chen, X. Ni, G. Liu, J. Huang and S. Peng, *J. Energy Chem.*, 2022, **64**, 23-32.
4 S. XI, X. CHENG, X. GAO and H. LIU, *Chinese Journal of Engineering*, 2024, **46**, 2036-2045.
5 S. Islam, M.H. Alfaruqi, V. Mathew, J. Song, S. Kim, S. Kim, J. Jo, J.P. Baboo, D.T. Pham and D.Y. Putro, *J. Mater. Chem. A*, 2017, **5**, 23299-23309.
6 I. Stoševski, A. Bonakdarpour, B. Fang, S.T. Voon and D.P. Wilkinson, *Int. J. Energy Res.*, 2021, **45**, 220-230.
7 L. Gou, K.L. Mou, X.Y. Fan, M.J. Zhao, Y. Wang, D. Xue and D.L. Li, *Dalton Trans.*, 2020, **49**, 711-718.
8 N. Zhang, F. Cheng, J. Liu, L. Wang, X. Long, X. Liu, F. Li and J. Chen, *Nat. Commun.*, 2017, **8**, 1-9.
9 J.W. Wang, Y.F. Yuan, D. Zhang, M. Zhu, C.L. Mo and S.Y. Guo, *Nanotechnology*, 2021, **32**, 435401.
10 J. Chen, L. Liao, B. Sun, X. Song, M. Wang, B. Guo, Z. Ma, B. Yu and X. Li, *J. Alloy. Compd.*, 2022, **903**, 163833.
11 Y. An, Y. Tian, Q. Man, H. Shen, C. Liu, Y. Qian, S. Xiong, J. Feng and Y. Qian, *ACS Nano*, 2022, **16**, 6755-6770.
